# Long-term surgical outcomes of gastric neuroendocrine carcinoma and mixed neuroendocrine-non-neuroendocrine neoplasms

**DOI:** 10.1186/s12957-022-02625-y

**Published:** 2022-05-24

**Authors:** Kenichi Iwasaki, Edward Barroga, Masaya Enomoto, Kazushige Tsurui, Yota Shimoda, Moe Matsumoto, Kenta Miyoshi, Yoshihiro Ota, Jun Matsubayashi, Yuichi Nagakawa

**Affiliations:** 1 Department of Gastrointestinal and Pediatric Surgery, Tokyo Medical University, 6-7-1 Nishishinjuku, Shinjuku-ku, Tokyo, 160-0023 Japan; 2 Graduate School of Nursing Science, St. Luke’s International University, Tokyo, Japan; 3 Department of Anatomic Pathology, Tokyo Medical University, Tokyo, Japan

**Keywords:** Gastric cancer, Neuroendocrine carcinoma, Mixed neuroendocrine-non-neuroendocrine neoplasms

## Abstract

**Background:**

Neuroendocrine carcinoma (NEC) and mixed neuroendocrine-non-neuroendocrine neoplasm (MiNEN) are extremely rare subtypes of gastric cancer. MiNEN is a mix of carcinomatous components and neuroendocrine neoplasm in the same lesion. NEC and MiNEN have a poor prognosis, are difficult to diagnose, and have no established treatment. Herein, we assessed the clinicopathological characteristics and long-term surgical outcomes of gastric NEC and MiNEN patients in our hospital.

**Methods:**

We retrospectively assessed 1538 patients pathologically diagnosed with gastric cancer and who underwent curative surgical resection at our institution between January 1999 and October 2021. Of these patients, 25 (1.6%) were pathologically diagnosed with neuroendocrine neoplasms. From these 25 patients, we retrospectively analyzed the clinicopathological characteristics and surgical outcomes of 13 (0.8%) patients pathologically diagnosed with NEC or MiNEN.

**Results:**

The NEC and MiNEN patients consisted of 11 men and 2 women [mean age, 74 (62–84) years]. The preoperative histological diagnoses were NEC (*n* = 4) and adenocarcinoma (*n* = 9). The final pathological diagnoses were large cell neuroendocrine carcinoma (LCNEC; *n* = 7) and MiNEN (*n* = 6). Total gastrectomy was the most common surgical procedure (9/13, 69.2%), followed by distal gastrectomy (3/13, 23.1%) and proximal gastrectomy (1/13, 7.7%). Immunohistochemical staining showed 8 CD56-positive patients. All 13 patients were positive for chromogranin A and synaptophysin. The mean Ki-67 value was 64.8 (0–95)%, and the mean mitotic score was 107.9 (0–400). Nine patients survived without recurrence postresection. The median postresection overall survival time was 68.7 (8.0–129) months. The 5-year survival rate was 0.75 ([95% CI] 0.408–0.912).

**Conclusion:**

The surgical treatment outcomes of NEC and MiNEN patients were relatively favorable. Although evidence concerning the effectiveness of surgery alone is meager, radical resection as part of multidisciplinary treatment including chemotherapy can potentially improve prognosis.

## Background

Neuroendocrine neoplasm (NEN) is a group of diseases that was previously called neuroendocrine tumor (NET). In the 2019 revision of the World Health Organization (WHO) classification, NET as a collective term was changed to NEN and NET was classified as a component of NEN [1]. NET and neuroendocrine carcinoma (NEC) are first distinguished according to the degree of histological differentiation into well-differentiated NET and poorly differentiated NEC. NEC is further classified into small cell type (SCNEC) and large cell type (LCNEC). In the 2019 WHO classification, epithelial tumors containing a mixture of NEN and non-NEN at a volume of 30% or more of the tumor for each component are defined as mixed neuroendocrine-non-neuroendocrine neoplasm (MiNEN).

Gastric NEC and MiNEN are extremely rare types of gastric cancer accounting for about 0.6% of all malignant gastric neoplasms [2–4]. NEC is associated with marked lymph node and liver metastases and is highly malignant. However, its classification, pathology, and treatment remain largely unclear. MiNEN’s biological behavior is mainly determined by its neuroendocrine component, and MiNEN may even have a worse prognosis and more aggressive behavior than NEC [5]. Because of the low incidence of gastric NEC and MiNEN, controversies remain regarding how to treat them and which treatment would be optimal. To date, few studies have been conducted to evaluate the necessity of radical resection for gastric NEC and MiNEN.

In this study, we assessed the clinicopathological characteristics and long-term surgical outcomes of gastric NEC and MiNEN patients in our hospital.

## Methods

We retrospectively reviewed all gastric cancer patients who underwent radical resection at Tokyo Medical University Hospital in Japan between January 1999 and August 2021. We obtained the clinicopathological characteristics and surgical outcomes of the patients from their medical charts, histological examinations, and pathology reports.

**Fig. 1:**
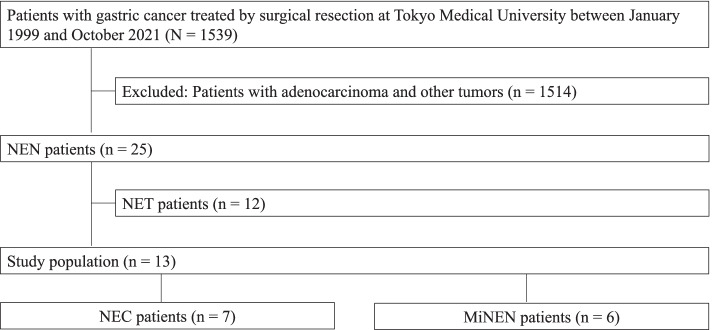
Flow diagram of patients registered in this study

We retrospectively analyzed the data of 25 consecutive patients who were pathologically diagnosed with gastric NEN. Of these patients, 13 patients who were pathologically diagnosed with gastric NEC (*n* = 7) or MiNEN (*n* = 6) were included in the present study. Patients who were diagnosed with well‑differentiated NETs were excluded. NEC and MiNEN were classified using the 5th edition of the 2019 WHO classification of tumors of the digestive system [1]. Figure 1 shows the flow diagram of the patients registered in the present study. Ki-67 index was calculated as the percentage of cells labeled by immunohistochemistry. Mitosis score was determined as the number of mitotic cells in 10 high-power fields.

### Ethics and informed consent

The Ethics Committee of Tokyo Medical University approved the study protocol (Approval No. T2019-0462) and waived the requirement for informed consent for the use of anonymized patient data. This study conforms to the provisions of the Declaration of Helsinki. All information was collected after obtaining written informed consent from the participants.

### Statistical analysis

Quantitative data were expressed as median and range. The Kaplan–Meier method was used to determine the cumulative survival rate. The log-rank test was applied to analyze the correlation between the clinicopathological factors and the survival of NEC and MiNEN patients. Statistical analysis was performed using SPSS 13.0. A *P*-value of < 0.05 was considered to indicate a statistically significant difference.

## Results

In this case series, there were 13 NEC and MiNEN patients (NEC, 7; MiNEN, 6) who received radical resection as a primary treatment. The clinical and surgical characteristics of the 7 NEC patients and 6 MiNEN patients are described in Tables 1 and 2, respectively. The total of 13 patients consisted of 2 women and 11 men with a mean age of 71 (63–78) years. According to the primary site of the tumor, 8 tumors were located in the upper third, 2 tumors in the middle third, and 3 tumors in the lower third. Regarding the macroscopic type, 3 tumors were superficial, 4 tumors were type Borrmann I, 2 tumors were Borrmann II, 3 tumors were Borrmann III, and 1 tumor was Borrmann IV. The mean maximum tumor diameter was 50.0 (15.0–107.0) mm.

**Table 1:** Clinicopathological characteristics of the neuroendocrine carcinoma (NEC) patients

Characteristics			*n* = 7
Age (years)	Median; range		73 (68–78)
Sex	Male		5
	Female		2
Body mass index (kg/m^2^)	Median; range		23 (19.4–30)
Performance status	0/1/2/3		5/2/0/0
Tumor location	Upper		5
	Middle		1
	Lower		1
Tumor diameter (mm)	Median; range		50 (15–107)
Macroscopic type	0/1/2/3/4/5		1/3/1/1/1/0
Surgical procedures	Distal gastrectomy		1
	Proximal gastrectomy		0
	Total gastrectomy		6
Extent of lymphadenectomy	Less than D2		4
	D2 or more		3
Tumor invasion	1/2/3/4		1/1/3/2
Lymph node metastasis	Negative		1
	Positive		6
Positive rate of lymph node metastasis (%)	Median; range		5.5 (0–21.5)
Component of positive lymph nodes	Neuroendocrine		7
	Non-neuroendocrine		0
	Both components		0
TNM stage	I/II/III/IV		1/3/3/0

**Table 2:** Clinicopathological characteristics of the mixed neuroendocrine-non-neuroendocrine neoplasm (MiNEN) patients

Characteristics		*n* = 6
Age (years)	Median; range	68 (63–74)
Sex	Male	6
	Female	0
Body mass index (kg/m^2^)	Median; range	23 (17.2–24)
Performance status	0/1/2/3	5/1/0/0
Tumor location	Upper	3
	Middle	1
	Lower	2
Tumor diameter (mm)	Median; range	45 (20–75)
Macroscopic type	0/1/2/3/4/5	2/1/1/2/0/0
Surgical procedures	Distal gastrectomy	2
	Proximal gastrectomy	1
	Total gastrectomy	3
Extent of lymphadenectomy	Less than D2	3
	D2 or more	3
Tumor invasion	1/2/3/4	2/1/0/3
Lymph node metastasis	Negative	3
	Positive	3
Positive rate of lymph node metastasis (%)	Median; range	4.4 (0–32.4)
Component of positive lymph nodes	Neuroendocrine	0
	Non-neuroendocrine	0
	Both components	3
TNM stage	I/II/III/IV	3/0/3/0

In the preoperative histological examination, 4 tumors were diagnosed as endocrine carcinoma and 9 tumors were diagnosed as adenocarcinoma. The final pathological diagnosis was large cell neuroendocrine carcinoma (LCNEC) in 7 patients and MiNEN in 6 patients. The accuracy of the preoperative diagnosis as to whether the tumor contained neuroendocrine carcinoma components was 30.7%. The pathological stage of the disease was IA in 3 patients, IB in 1 patient, IIB in 3 patients, IIIA in 2 patients, IIIB in 2 patients, and IIIC in 2 patients. Total gastrectomy was the most common surgical procedure (9/13, 69.2%), followed by distal gastrectomy (3/13, 23.1%) and proximal gastrectomy (1/13, 7.7%). Lymphadenectomy and curative surgical resection were performed in all cases. Postoperative adjuvant chemotherapy was performed in 5 (38.5%) cases.

The results of the immunohistochemistry analysis are summarized in Table 3. Immunohistochemically, 8 cases of the 13 NENs were positive for CD56. Twelve cases (92.3%) were positive for chromogranin A and all NENs were positive for synaptophysin. The mean Ki-67 labeling index was 64.8 (0–95)%, the mean mitotic score was 107.9 (0–400), lymphatic vessel invasion was positive in 11, and venous invasion was positive in 8 patients.

**Table 3:** Immunohistochemical findings

Histological type (2019 WHO classification)	
SCNEC	0
LCNEC	7
MiNEN	6
Lymphatic invasion	
0/1/2/3	2/6/3/2
Vascular invasion	
01/2/3	5/1/4/3
Coexistence of adenocarcinoma	
Absent	4
Present	9
Neuroendocrine markers	
CD56	
Positive/negative	8/5
Chromogranin A	
Positive/negative	12/1
Synaptophysin	
Positive/negative	13/0
Differentiation	
Mitotic score	
Median; range	66 (0–400)
Ki-67 index %	
Median; range	60 (0–95)

*SCNEC*, small cell neuroendocrine carcinoma; *LCNEC*, large cell neuroendocrine carcinoma; *MiNEN*, mixed neuroendocrine-non-neuroendocrine neoplasm

The treatments and outcomes of the NEC and MiNEN patients are summarized in Table 4. All patients underwent curative surgery. The median follow-up period was 68.7 months (range 8.0–129). Recurrence occurred in 4 patients (30.8%) who underwent curative surgery. Neither of the cases who showed recurrence received adjuvant chemotherapy. Nine patients survived without recurrence after resection. The 5-year recurrence-free survival (RFS) rate was 0.65 ([95% CI] 0.310–0.852). The median postoperative overall survival (OS) time in all the resected cases was 68.7 (8.0–129) months, and the 5-year survival rate was 0.75((95% CI) 0.408–0.912).

**Table 4:** Treatments and outcomes of the NEC and MiNEN patients

Patient	Diagnosis	Surgical procedure	pStage	NAC	Adjuvant chemotherapy	REC	OS (months)	RFS (months)	Outcome	Cause of death
1	LCNEC	TG	IIB	None	S-1	None	93.8	93.8	Alive	-
2	MiNEN	TG	IA	None	None	None	99.9	99.9	Alive	-
3	LCNEC	TG	IIB	None	None	liver	37.3	6.9	Dead	Cancer
4	MiNEN	DG	IIIB	None	S-1	None	99.7	99.6	Alive	-
5	LCNEC	TG	IIIC	None	None	LN	63.7	5.4	Dead	Cancer
6	LCNEC	TG	IIB	None	S-1	None	129	129	Alive	-
7	LCNEC	DG	IA	None	None	None	24.6	24.6	Alive	-
8	MiNEN	TG	IB	None	None	none	67.4	67.4	Alive	-
9	MiNEN	TG	IIIA	none	None	P	39.9	18.2	Dead	Cancer
10	LCNEC	TG	IIIA	None	S-1	None	68.7	68.7	Alive	-
11	MiNEN	PG	IA	None	None	None	77.3	77.3	Alive	-
12	MiNEN	DG	IIIC	None	None	P	72.1	6.67	Dead	Cancer
13	LCNEC	TG	IIIB	None	CDDP + CPT-11	None	12	12	Alive	-

*LCNEC*, large cell neuroendocrine carcinoma; *MiNEN*, mixed neuroendocrine-non-neuroendocrine neoplasm; *TG*, total gastrectomy; *DG*, distal gastrectomy; *PG*, proximal gastrectomy; *S-1*, Tegafur/gimeracil/oteracil; *CDDP*, cisplatin; *CPT-11*, irinotecan; *pStage*, pathological stage; *NAC*, neoadjuvant chemotherapy; *REC*, recurrence; *OS*, overall survival; *RFS*, recurrence-free survival; *LN*, lymph nodes; *P*, peritoneum

**Fig. 2:**
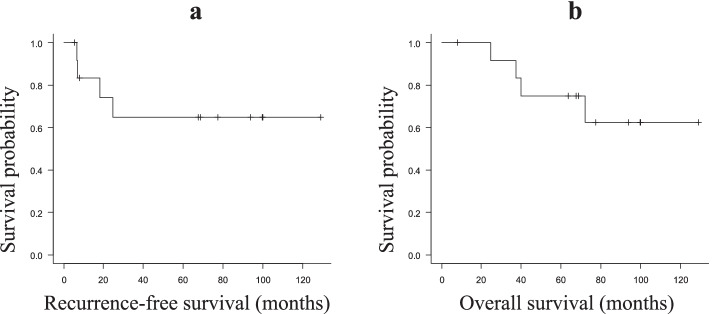
**a** Recurrence-free survival (RFS) curves of patients with NEC and MiNEN (*n* = 13). **b** Overall survival (OS) curves of patients with NEC and MiNEN (*n* = 13)

**Fig. 3:**
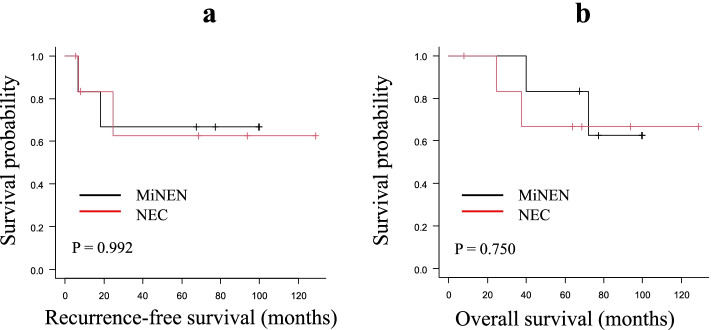
**a** Recurrence-free survival (RFS) curves of NEC patients (*n* = 7) and MiNEN patients (*n* = 6). There was no significant difference in the RFS (*p* = 0.992). **b** Overall survival (OS) curves of NEC patients (*n* = 7) and MiNEN patients (*n* = 6). There was no significant difference in the OS (*p* = 0.992)

The Kaplan–Meier survival curves for RFS and OS are shown in Fig. 2a and b. Regarding the survival analyses shown in Fig. 3a and b, there was no significant difference in either the recurrence-free period or the overall survival period (*P* = 0.992, *P* = 0.75) between the NEC patients and the MiNEN patients. Table 5 shows the univariate and multivariate Cox proportional hazard analyses of the 11 prognostic factors (e.g., age, tumor diameter, pathological type) in the model. There was no clinicopathological factor associated with the survival after the surgical resection of NEC and MiNEN.

**Table 5:** Univariate and multivariate Cox proportional hazard analyses of clinicopathological factors for survival

Characteristics		Number	Univariate			Multivariate		
			**OR**	**95%CI**	***P*-value**	**OR**	**95% CI**	***P*-value**
Age (y/o)	< 70	6	1.000	0.132–0.334	0.215	-	-	-
	≥ 70	7	1.493					
Gender	Female	2	1.000	0.508–0.987	0.583	-	-	-
	Male	1	1.342					
Tumor diameter (mm)	< 35	8	1.000	0.081–12.360	0.897	-	-	-
	≥ 35	5	1.003					
Pathological type	LCNEC	7	1.000	0.100–5.240	0.751	-	-	-
	MiNEN	6	0.726					
T stage	T1-T2	5	1.000	0.429–6.140	0.583	-	-	-
	T3-T4	8	1.408					
Lymph node metastasis	Negative	4	1.000	0.438–2.092	0.750	-	-	-
	Positive	9	1.218					
Lymphatic invasion	Negative	2	1.000	0.744–7.211	0.478	-	-	-
	Positive	1	1.332					
Vascular invasion	Negative	5	1.000	0.102–5.616	0.837	-	-	-
	Positive	8	1.274					
Ki-67 index %	< 60	5	1.000	0.266–4.074	0.897	-	-	-
	≥ 60	8	1.193					
Surgical procedures	Total gastrectomy	9	1.000	0.228–3.041	0.427	-	-	-
	Distal/proximal gastrectomy	4	1.038					
Adjuvant chemotherapy	Yes	5	1.000	0.846–21.129	0.100	-	-	-
	No	8	1.940					

## Discussion

It has been reported that NEC and MiNEN are both associated with aggressive oncological behavior, extensive lymph node dissemination, high invasiveness, and poor prognosis [6]. However, the rarity and poor prognosis of both NEC and MiNEN make it difficult to understand their biological nature and establish a standard treatment. In fact, in the present study, we found an extremely rare frequency of NEC and MiNEN at 0.8% in gastric cancer patients who underwent surgical resection. The present study is thus a valuable addition to the scarce studies of both tumors, aiming at clarifying the survival outcomes of patients following the surgical resection of gastric neoplasms composed of neuroendocrine components (NEC and MiNEN).

A common treatment strategy for NEC and MiNEN based on the Japanese Classification of Gastric Carcinoma is a combination of classic surgery and adjuvant chemotherapy [7]. However, there are still no clear clinical guidelines or consensuses that have been developed. Regarding chemotherapy, there is still no regimen that has been established. The National Comprehensive Cancer Network guidelines recommend basically similar treatments as those for small cell lung cancer. On the other hand, the European Neuroendocrine Tumor Society guidelines recommend CDDP + ETP. For operative treatment, surgical resection with lymphadenectomy is the standard treatment. However, chemotherapy alone is often elected because of the rapid progression of the disease.

It has been reported that NEC and MiNEN are immunohistochemically positive for synaptophysin and chromogranin A, invade blood vessels from an early stage, metastasize to multiple organs, and have a poor prognosis [2, 8–10]. However, the physiopathology of NEC and MiNEN remains uncertain. There are two major hypotheses regarding the pathogenic mechanism of gastric NEN. The first hypothesis is that part of adenocarcinoma that has infiltrated the submucosal layer differentiates into endocrine progenitor cells, which then form NEC or MiNEN [11]. The second hypothesis is that monoclonal multipotent epithelial stem cells differentiate into two components [12]. The second hypothesis is likely to be more accepted at present in view of recent findings [13]. This complex pathogenic mechanism makes the diagnosis of neuroendocrine tumors difficult, with only a preoperative diagnostic rate of 30% [14].

According to the findings of Ma et al. [6], neoadjuvant chemotherapy (NAC) may provide survival benefits for patients with gastric cancer having neuroendocrine components. Although none of our 13 patients received NAC, this is based on a fact that NEC and MiNEN tend to be misdiagnosed as adenocarcinoma by biopsy assessments. Similarly to the findings of Uematsu et al. [14], only 4 cases (30.8%) in our case series were histologically diagnosed as endocrine carcinoma by preoperative biopsy. The diagnosis and early detection of NEC and MiNEN are usually crucial but difficult. Both NEC and MiNEN show nonspecific gastric cancer symptoms and are usually diagnosed after surgery (in an advanced disease stage) when sufficient specimen is obtained. If NAC becomes the mainstream treatment of NEC and MiNEN in the future, immunohistochemical analysis for differential diagnosis may need to be reconsidered carefully in clinical practice.

More than half of the patients with a gastric tumor consisting of a neuroendocrine component reportedly show a short OS of less than 12 months and a mean survival period of 9.3–15 months [15, 16]. MiNEN may even have a worse prognosis compared with NEC [5]. On the other hand, adjuvant chemotherapy may play an important role in the survival of patients with NEC and MiNEN [6, 17]. Interestingly in the present study, all the patients who showed tumor recurrence after surgical resection did not receive adjuvant chemotherapy. In our case series, univariate and multivariate Cox proportional hazard analyses of the clinicopathological factors associated with survival after surgical resection of NEC and MiNEN showed no independent prognostic factors including adjuvant chemotherapy.

The prognosis of NEC is poor because of its marked vascular invasion and distant metastasis from the early stages and the absence of an effective secondary treatment. However, Ishida et al. observed that the median OS rate of NEC patients was 46 months and that the prognosis of NEC is not necessarily poorer than that of adenocarcinoma [18]. In the present study, the 13 surgically resected NEC and MiNEN cases showed a relatively good outcome. Nine patients survived without recurrence after resection. The median postoperative OS time in all the resected cases was 68.7 (7.93–129) months, and the 5-year survival rate was 75.0% (95% CI, 40.8–91.2). Relapse-free survival was achieved in 8 patients (61.5%). These findings indicate the beneficial roles of radical resection when possible. However, in many NEC and MiNEN cases, remote metastasis has already occurred at the time of diagnosis; therefore, chemotherapy is performed as a primary treatment in many cases.

There is presently no gold standard of treatment for gastric NEC and MiNEN, and they are often treated as gastric cancer. Based on our findings, optimal surgical resection procedures followed by adjuvant chemotherapy may provide better outcomes.

Our study has several limitations. First, the analysis used a retrospective nonrandomized design with a small sample size. This limited number of patients was due to the rareness of NEC and MiNEN cases. Nevertheless, our sample size is not smaller than that of previous reports. Second, there was no patient with SCNEC. This was likely due to the small sample size, which could have led to some biases. Third, given that all our patients underwent surgery, there is no comparison group with a similar background that did not undergo surgery. A large-scale prospective multicenter study must be conducted to further clarify NEC and MiNEN and accumulate data for the development of future therapeutic strategies.

## Conclusion

This study showed that the long-term survival outcomes of patients undergoing surgical resection of gastric NEC and MiNEN were acceptable in terms of a favorable disease-free progression and mortality. NEC and MiNEN are extremely rare diseases, and the prediction of prognostic factors based on their histological characteristics is expected to become feasible with further accumulation and clarification of cases.

## Data Availability

The datasets generated and/or analyzed during the current study are available from the corresponding author upon reasonable request.

## Abbreviations

NEC: Neuroendocrine carcinoma
MiNEN: Mixed neuroendocrine-non-neuroendocrine neoplasms
LCNEC: Large cell neuroendocrine carcinoma
SCNEC: Small cell neuroendocrine carcinoma
NAC: Neoadjuvant chemotherapy
NEN: Neuroendocrine neoplasm
NET: Neuroendocrine tumor
RFS: Recurrence-free survival
